# Metabarcoding of the phytotelmata of *Pseudalcantarea grandis* (Bromeliaceae) from an arid zone

**DOI:** 10.7717/peerj.12706

**Published:** 2022-01-27

**Authors:** José Alan Herrera-García, Mahinda Martinez, Pilar Zamora-Tavares, Ofelia Vargas-Ponce, Luis Hernández-Sandoval, Fabián Alejandro Rodríguez-Zaragoza

**Affiliations:** 1 Universidad Autónoma de Querétaro, Querétaro, Mexico; 2 Laboratorio Nacional de Identificación y Caracterización Vegetal, Querétaro, Mexico; 3 Instituto de Botánica, departamento de Botánica y Zoología, Centro Universitario de Ciencias Biológicas y Agropecuarias, Universidad de Guadalajara, Guadalajara, Jalisco, México; 4 Laboratorio Nacional de Identificación y Caracterización Vegetal, Guadalajara, Mexico; 5 Laboratorio de Ecología Molecular, Microbiología y Taxonomía (LEMITAX), Departamento de Ecología, Centro Universitario de Ciencias Biológicas y Agropecuarias, Universidad de Guadalajara, Guadalajara, Jalisco, Mexico

**Keywords:** Bromeliads, High endemicity, Water sample, 16S, Proteobacteria, Actinobacteria, Firmicutes, Taxonomic variation, Functional redundancy, Resilience

## Abstract

**Background:**

*Pseudalcantarea grandis* (Schltdl.) Pinzón & Barfuss is a tank bromeliad that grows on cliffs in the southernmost portion of the Chihuahuan desert. Phytotelmata are water bodies formed by plants that function as micro-ecosystems where bacteria, algae, protists, insects, fungi, and some vertebrates can develop. We hypothesized that the bacterial diversity contained in the phytotelma formed in a bromeliad from an arid zone would differ in sites with and without surrounding vegetation. Our study aimed to characterize the bacterial composition and putative metabolic functions in *P. grandis* phytotelmata collected in vegetated and non-vegetated sites.

**Methods:**

Water from 10 individuals was sampled. Five individuals had abundant surrounding vegetation, and five had little or no vegetation. We extracted DNA and amplified seven hypervariable regions of the 16S gene (V2, V4, V8, V3–6, 7–9). Metabarcoding sequencing was performed on the Ion Torrent PGM platform. Taxonomic identity was assigned by the binning reads and coverage between hit and query from the reference database of at least 90%. Putative metabolic functions of the bacterial families were assigned mainly using the FAPROTAX database. The dominance patterns in each site were visualized with rank/abundance curves using the number of Operational Taxonomic Units (OTUs) per family. A percentage similarity analysis (SIMPER) was used to estimate dissimilarity between the sites. Relationships among bacterial families (identified by the dominance analysis and SIMPER), sites, and their respective putative functions were analyzed with shade plots.

**Results:**

A total of 1.5 million useful bacterial sequences were obtained. Sequences were clustered into OTUs, and taxonomic assignment was conducted using BLAST in the Greengenes databases. Bacterial diversity was 23 phyla, 52 classes, 98 orders, 218 families, and 297 genera. Proteobacteria (37%), Actinobacteria (19%), and Firmicutes (15%) comprised the highest percentage (71%). There was a 68.3% similarity between the two sites at family level, with 149 families shared. Aerobic chemoheterotrophy and fermentation were the main metabolic functions in both sites, followed by ureolysis, nitrate reduction, aromatic compound degradation, and nitrogen fixation. The dominant bacteria shared most of the metabolic functions between sites. Some functions were recorded for one site only and were related to families with the lowest OTUs richness. Bacterial diversity in the *P. grandis* tanks included dominant phyla and families present at low percentage that could be considered part of a rare biosphere. A rare biosphere can form genetic reservoirs, the local abundance of which depends on external abiotic and biotic factors, while their interactions could favor micro-ecosystem resilience and resistance.

## Introduction

Phytotelmata are water bodies formed by plants that function as micro-ecosystems (Benzing, 2000). The community comprises bacteria, cyanobacteria, protists, fungi, green algae, mosses, vascular plants, insects, crustaceans, and a few vertebrates (Benzing, 2000; Kitching, 2001; Brandt, Martinson & Conrad, 2016). Under natural conditions, organisms are frequently replaced, and the system has been used as a study model for food webs (Mogi, 2004). Phytotelmata are most frequently found in tropical areas but can also occur in temperate forests, swamps, and deserts. In arid environments, the phytotelma-associated micro-ecosystem is defined by the seasonality of water availability. Once water accumulates following the rains, growth occurs in the aquatic biota that is well adapted to temporary environments, significantly increasing the diversity of aquatic organisms in the area (Calhoun et al., 2017).

Although different plant families form phytotelmata, the Bromeliaceae have various anatomical, morphological, and physiological adaptations that allow them to grow in areas with wide resource variations (Giongo et al., 2019). For example, the leaves are arranged in a tight rosette, and the plant epidermis is covered with trichomes that absorb humidity and nutrients, allowing the plants to grow in arid environments with scarce nutrients (Benzing, 2000; Goffredi, Kantor & Woodside, 2011). *Pseudalcantarea grandis* (Schltdl.) Pinzón & Barfuss is a bromeliad found in saxicolous habitats, up to 2.5 m in height and with a branched inflorescence present in March and April. It is native to central-eastern Mexico to Honduras (Rzedowski, 2006). The species thrives on canyon cliffs of the major rivers of the northeastern Bajío region, Mexico, at altitudes ranging from 400 to 1,600 m asl. Due to the inaccessibility of its populations, it presents no particular conservation problems.

Characterization and identification of organisms contained in environmental samples can be conducted using different approaches, such as sample culture, target sequencing, metabarcoding, metatranscriptomics, and metagenomics. The diversity of specific groups in the tank bromeliads has been analyzed with targeted sequencing on ciliates and vertebrates (Brozio et al., 2017; Simão et al., 2017). Using metatranscriptomics, Goffredi, Jang & Haroon (2015) found 450 species of Archaea and bacteria in *Vriesea platynema* Gaudich. (Bromeliaceae) tanks. Metabarcoding is the direct analysis of DNA fragments contained in an environmental sample (Cabral et al., 2018). This technique allows the identification of microorganisms with no need for culturing (Rodríguez-Nuñez, Rullan-Cardec & Rios-Velazquez, 2018). Metabarcoding has been used to identify bacterial and eukaryotic biodiversity in the phytotelmata of *Sarracenia purpurea* L. (Sarrraceniaceae) (Grothjan & Young, 2019). In tank bromeliads, bacterial metabarcoding has been used in five studies, four in Brazil and one in Puerto Rico, all in tropical forests (Louca et al., 2016; Louca et al., 2017; Simão et al., 2017; Rodríguez-Nuñez, Rullan-Cardec & Rios-Velazquez, 2018; Giongo et al., 2019; Simão et al., 2020). To our knowledge, however, arid zone bromeliads have not been studied.

The biotic composition of the phytotelmata depends on the species, its location, and local factors that affect water conditions (Benzing, 2000; Louca et al., 2016; Louca et al., 2017; Males, 2016). Bromeliad tanks form a unique freshwater environment that differs in oxygen concentration and pH from the external environment, thus providing a habitat for a diverse community (Goffredi, Kantor & Woodside, 2011). The phytotelmata in bromeliads from tropical forests can contain methanogens, which are microorganisms responsible for carbon cycling (Goffredi, Kantor & Woodside, 2011). When comparing the community of archaea and methanogens in phytotelmata from different tank water volume, it was found that the methane cycle formation in the phytotelma decreases during dry periods in neotropical forests (Brandt, Martinson & Conrad, 2016). Identifying the bacterial communities of bromeliad phytotelmata from different ecological niches can help to understand their interaction with the metabolism of the host plant (Louca et al., 2017). The phytotelmata of *P. grandis* constitutes a temporary aquatic ecosystem in a desert, and its biodiversity has not been studied. Although water availability is highly seasonal, we hypothesized that the tank bacterial composition will differ in sites with and without surrounding vegetation. Our study aimed to characterize the bacterial composition and putative metabolic functions in *P. grandis* phytotelmata collected in vegetated and non-vegetated sites.

## Materials and Methods

### Site descriptions, plant selection, and sampling

**Figure 1:**
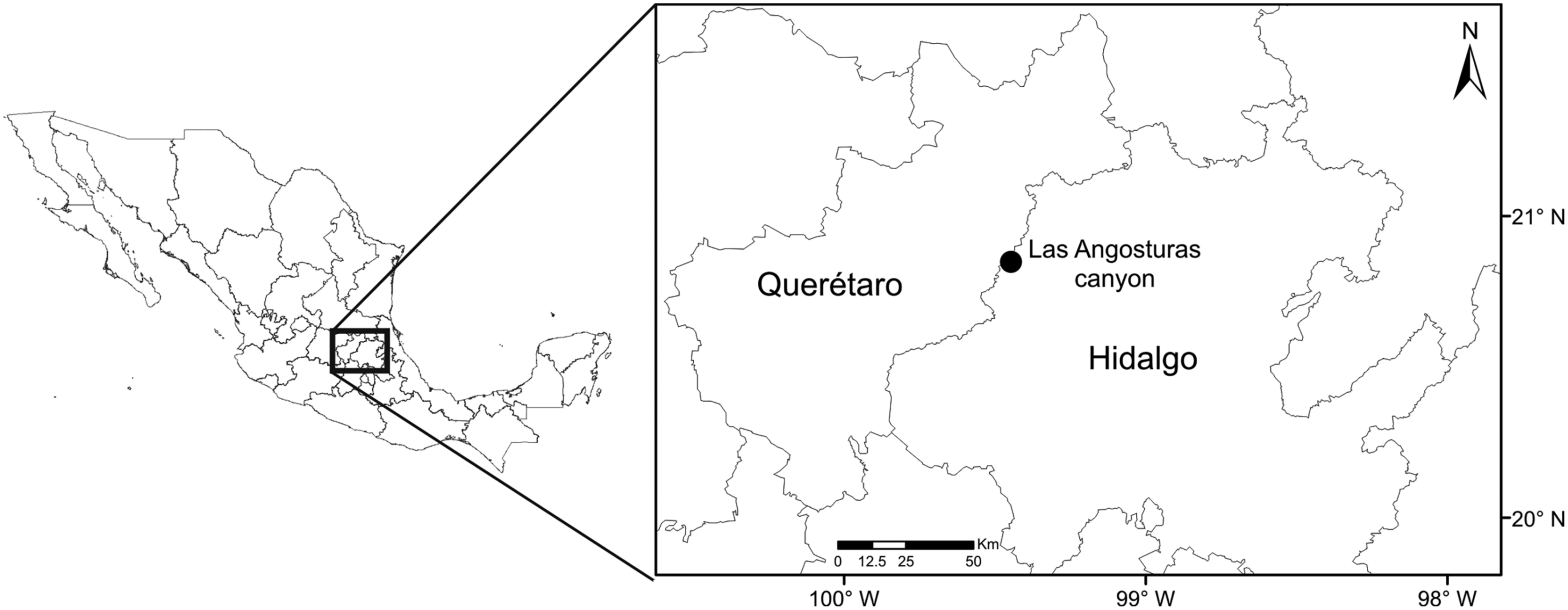
Collection site map.

**Figure 2:**
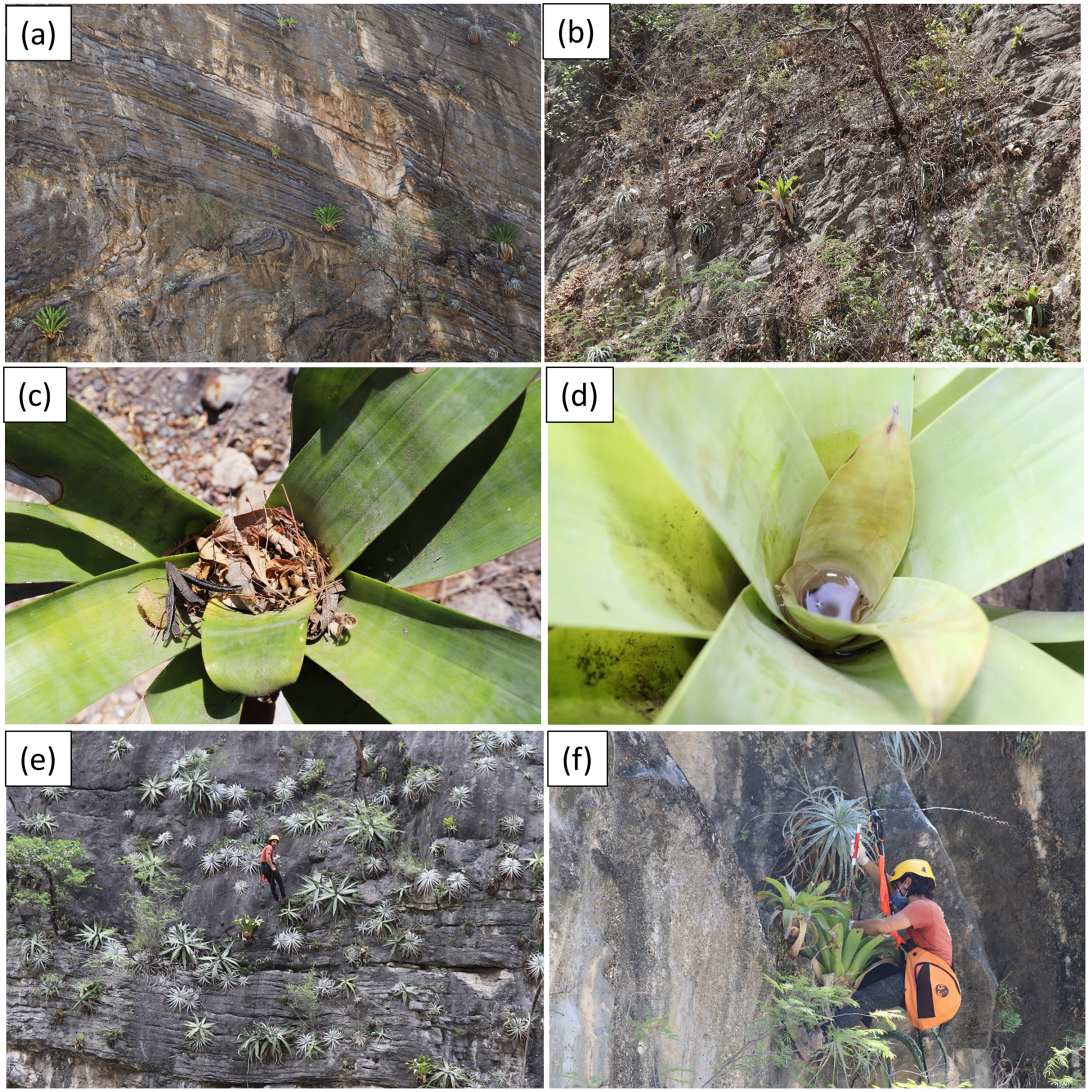
Vegetated and unvegetated sites. (A) Little or no surrounding vegetation. (B) Abundant surrounding vegetation. (C) Phytotelmata dry and full of debris. (D) Phytotelmata with water and no debris. (E & F) Rapel sampling.

The study site is located in the Las Angosturas canyon, also known as Barranca Tolimán, in Zimapán, Hidalgo, in central Mexico (20°50.933′N, 99°26.7′W, 900 masl) (Fig. 1). The area is located in the southernmost portion of the Chihuahuan desert (Hernández & Gómez-Hinostrosa, 2005) and constitutes a local floristic region of high endemism (Medellín-Leal, 1982). The exact location of the study area does not feature in any geomorphological or geological publications. However, adjacent canyons in the same region have been subjected to detailed studies (Segestrom, 1961; Carrillo, 1981; Carrillo & Sutter, 1981; Arévalo, 1991). The geological formations are Trancas (Late Jurassic, Early Cretaceous), el Doctor (Middle Cretaceous), and Soyatal (Upper Cretaceous), formed by a combination of calcareous rocks alternated with calcareous limestones, calcareous lutites, and sandstones. Structurally, the canyon is formed by rocky vertical cliffs at 80–90° angles. The *P. grandis* plants grow on sandstone rocks on the vertical cliffs (Fig. 2) of the El Doctor formation. Ten individuals of 50 cm or more in diameter were sampled on cliffs: five with little or no vegetation (Fig. 2A) and five with abundant surrounding vegetation (Fig. 2B). Four of our vegetated sample sites had a NE orientation and one a NW orientation; all sites were surrounded by either xerophytic scrub or tropical deciduous forest. The non-vegetated sites all faced N. The plant species surrounding the sample sites were identified and recorded (Table 1). Water samples were collected in June 2018 during the rainy season since the plants are dry for the rest of the year, either empty or full of debris (Figs. 2C, 2D). Experiments were approved by the “Comité de Bioética de la Facultad de Ciencias Naturales” bioethics committee (39FCN2019). Bromeliads were reached by rappel (Figs. 2E, 2F). Nest® cell scrapers were used to scratch the inside of each tank, and the water in the bromeliad was vigorously shaken in order to obtain a homogeneous sample. Water volumes of 50 to 100 ml were collected using 10 ml sterile serological pipettes. Samples were stored in 50 ml conical Falcon tubes, transported on dry ice, and stored at −79 °C until processed.

**Table 1:** Floristic inventory at Las Angosturas canyon.

Family	Scientific name	Vegetated/unvegetated site
Asparagaceae	*Agave xylonacantha* Salm-Dyck	Vegetated/unvegetated
Asteraceae	*Gochnatia hypoleuca* (DC.) A. Gray	Vegetated
Bromeliaceae	*Hechtia glomerata* Zucc.	Vegetated
Bromeliaceae	*Hechtia tillandsioides* (André) L. B. Smith	Vegetated/unvegetated
Bromeliaceae	*Tillandsia recurvata* L.	Vegetated
Burseraceae	*Bursera morelensis* Ram.	Vegetated
Cactaceae	*Mammilaria elongata* DC.	Vegetated
Cactaceae	*Myrtillocactus geometrizans* (Mart. ex Pfeiff.) Console	Vegetated
Cactaceae	*Opuntia rastrera* F.A.C. Weber	Vegetated
Crassulaceae	*Echeveria secunda* Booth	Vegetated
Crassulaceae	*Sedum*	Vegetated
Fabaceae	*Acacia berlandieri* Benth.	Vegetated
Fabaceae	*Mimosa leucaenoides* Bentham	Vegetated
Fouquieriaceae	*Fouquieria splendens* Engelm.	Vegetated
Onagraceae	*Hauya elegans* DC.	Vegetated
Myrtaceae	*Psidium guajava* L.	Vegetated
Selaginellaceae	*Selaginella lepidophylla* (Hook. & Grev.) Spring.	Vegetated
Zygophyllaceae	*Morkillia acuminata* Rose & Painter	Vegetated

### DNA extraction and sequencing

The five samples of each site were homogenized and pooled. A total of 100 ml of sampled water was filtered through a 0.22 µm nitrocellulose Millipore® membrane. The membrane was then frozen and macerated in liquid nitrogen. DNA was extracted in triplicate with the QIAmp DNA extraction® kit following the manufacturer’s instructions. DNA quality and quantity were evaluated using spectrophotometry in a NanoDrop® instrument. PCR amplicons of seven hypervariable regions of the 16S gene were amplified with two primer sets, the first targeting V2, V4, V8, and the second V3–6, 7–9, with the Ion 16S™ Metagenomics kit (Thermo Fisher Scientific, Waltham, MA, USA), following the manufacturer’s protocol. The metabarcoding sequencing was performed on the Ion Torrent PGM platform, and the amplicons were purified with Agencourt® AMPure® XP. The Ion Plus Fragment Library kit protocol was followed in order to construct the libraries. Fragment presence, size, and concentration were analyzed using a Bioanalyzer 2100 with the High Sensitivity DNA assay (Agilent, Santa Clara, CA, USA). Libraries were quantified using real-time PCR to obtain an equimolar dilution factor for mixing the libraries. Templates were prepared *via* an emulsion PCR in the Ion One Touch System (Life Technologies, Carlsbad, CA, USA) and quantified in a fluorometer in Qubit® 3.0 (Thermo Fisher Scientific, Waltham, MA, USA). The template was loaded in the PGM 318™ chip using the sequencing kit for 400 base pairs, following the Ion PGM™ Hi‑Q™ View Sequencing Kit protocol.

### Data analysis

#### Bioinformatic analysis

Bacteria were determined using Ion Reporter™. Sequencing results were analyzed using the metagenomics application for multiple groups based on the Greengenes v13.5 database. Primers used for amplification were identified, and a minimum sequence length of 150 bp was defined. To assign taxonomic identity, we considered two criteria: the binning reads had to be repeated at least 10 times, and the coverage between hit and query from the reference database had to be at least 90%.

#### Analysis of bacterial composition between sites

Bacterial families were ordered by taxonomic hierarchy for each site, and a richness stacked barplot was produced at order and family level with Microsoft Excel tools. The bacterial composition of the two sites vegetated (V) and non-vegetated (NV) was compared using the Sørensen similarity coefficient based on a presence/absence matrix for bacterial families, and a Venn diagram was generated using vegan and VennDiagram packages in R Studio v3.6.1 (R Core Team, 2019). In addition, the dominance patterns of bacterial families were visualized with rank/abundance curves, using the number of OTUs per family. A percentage similarity analysis (SIMPER) was used to estimate the dissimilarity between sites. SIMPER was performed with the composition and number of OTUs per family, a data pretreatment by square root-transformation, and the Bray–Curtis similarity coefficient.

A shade plot was constructed using the most important bacterial families, according to dominance and contribution to the dissimilarity between the vegetated and non-vegetated sites. These bacterial families were selected with the bacterial dominance analysis and SIMPER results, considering a cumulative contribution of ~40% in both. In this shade plot, a matrix of family composition and number of OTUs was used. For the classification of families, a Whittaker association coefficient was used with data previously standardized to percentages, and the group average linkage method. In the samples from vegetated and non-vegetated sites, a Bray-Curtis similarity and a square-root transformation were used.

#### Metabolic functions

To identify putative metabolic functions, we used the FAPROTAX database v.1.2.4 (Louca, Parfrey & Doebeli, 2016). This database assigns a putative metabolic function to each OTU based on the literature and, for some taxa, associates this function with cultured taxa with a verified function in the same taxonomic group. The current bacterial diversity not recognized under culture is high, and therefore the generalized assignment may change in future studies. However, this database provides information on 4,600 taxa (Louca et al., 2017). We analyzed the data in two ways: combined and separated (vegetated and non-vegetated sites), and with data from each site separately. Function was assigned at the family and genus level whenever possible. The putative taxa function that was absent from the FAPROTAX database was inferred based on the available literature. We looked for the family name and then reviewed its metabolic functions (Bergey & Holt, 2005; Louca et al., 2016; Louca et al., 2017). Furthermore, the relationship between bacterial families identified by the dominance and SIMPER analysis, and their respective putative functions, was analyzed with another shade plot. This analysis was performed with a binary matrix based on the Sørensen similarity coefficient to associate families and functions using the group average linkage method. The range/abundance curves, SIMPER, and shade plots were generated in PRIMER 7 7.0.21 (Clarke & Gorley, 2015).

## Results

**Figure 3:**
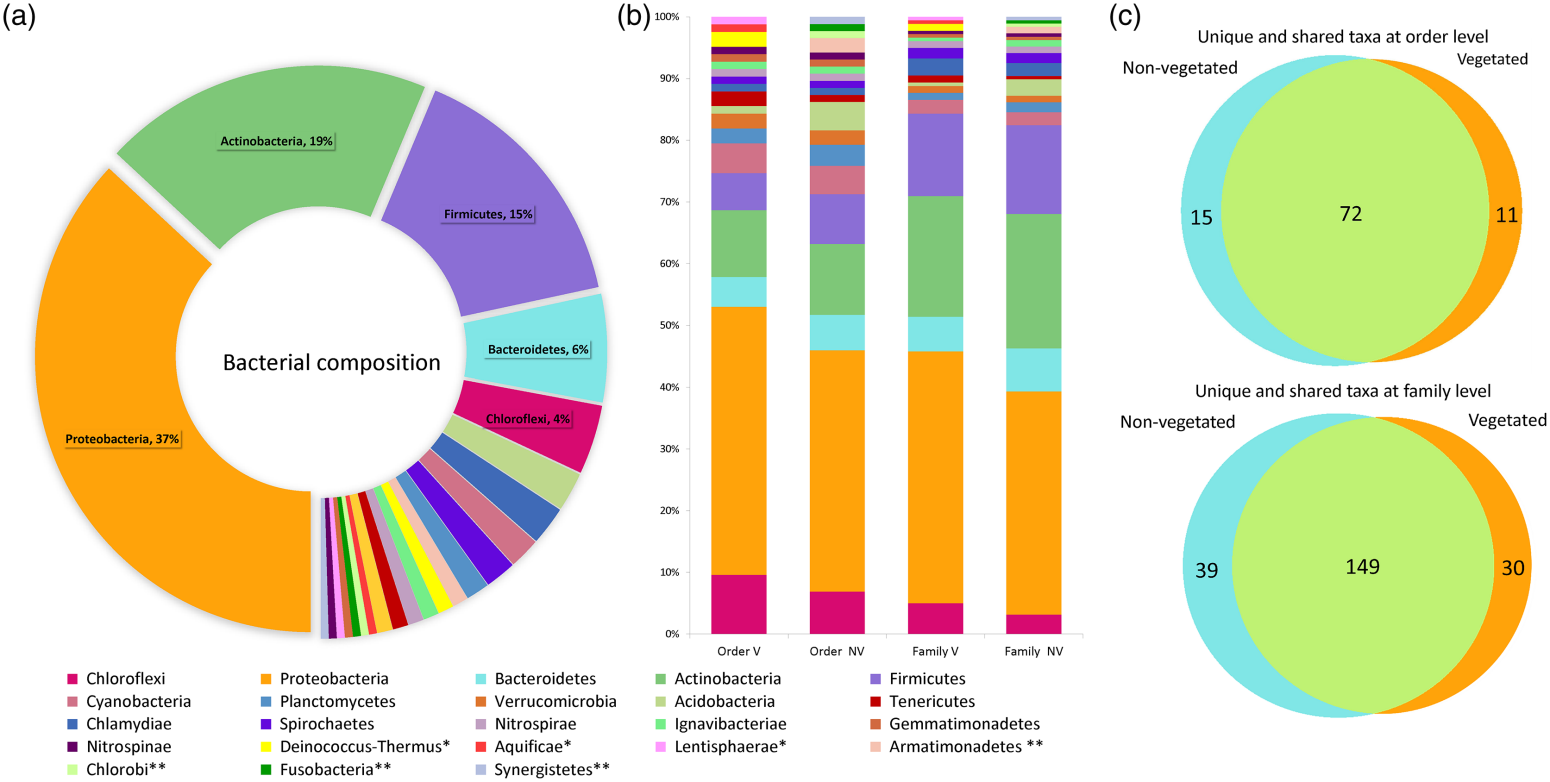
Percentage of relative abundance present in the *Pseudoalcantarea grandis* tank. Relative abundance of bacterial taxa present in the *Pseudoalcantarea grandis* tank. (A) Bacterial composition and abundance at phylum level of all bromeliads samples. (B) Community composition at order and family level at vegetated (V) and non-vegetated (NV) sites *taxa exclusive at the vegetated site, **taxa exclusive at the non-vegetated site. (C) Venn diagram showing the unique and shared taxa at order and family level.

The water volume of each bromeliad varied from 50 to 150 ml. A total of 5,411,296 reads was obtained. Once depurated, 1,499,606 sequences were considered useful, constituting 208,306 binning reads within the phytotelma. The bacterial dataset included 23 phyla, 52 classes, 98 orders, 218 families, and 297 genera (Table S1). Three phyla comprised the highest percentage of the bacterial community: Proteobacteria (37%), Actinobacteria (19%), and Firmicutes (15%). The remained 29% comprised the phyla Acidobacteria, Aquifica, Armatimonadetes, Bacteroidetes, Chlamydiae, Chlorobi, Chloroflexi, Cyanobacteria, *Deinococcus-Thermus*, Fusobacteria, Gemmatimonadetes, Ignavibacteriae, Lentisphaerae, Nitrospinae, Nitrospirae, Planctomycetes, Spirochaetes, Synergistetes, Tenericutes, and Verrucomicrobia, ranging from 5.8 to 0.5% (Fig. 3A). The phytotelmata of *P. grandis* in the vegetated site contained 19 phyla, 41 classes, 83 orders, and 179 families, 30 of which were exclusive. In the non-vegetated site, 20 phyla, 44 classes, 87 orders, and 188 families were found, and 39 families were exclusive (Fig. 3B). The Sørensen coefficient indicates a 68.3% similarity between the two sites at the family level, with 149 of the 218 families shared between both (Fig. 3C).

**Figure 4:**
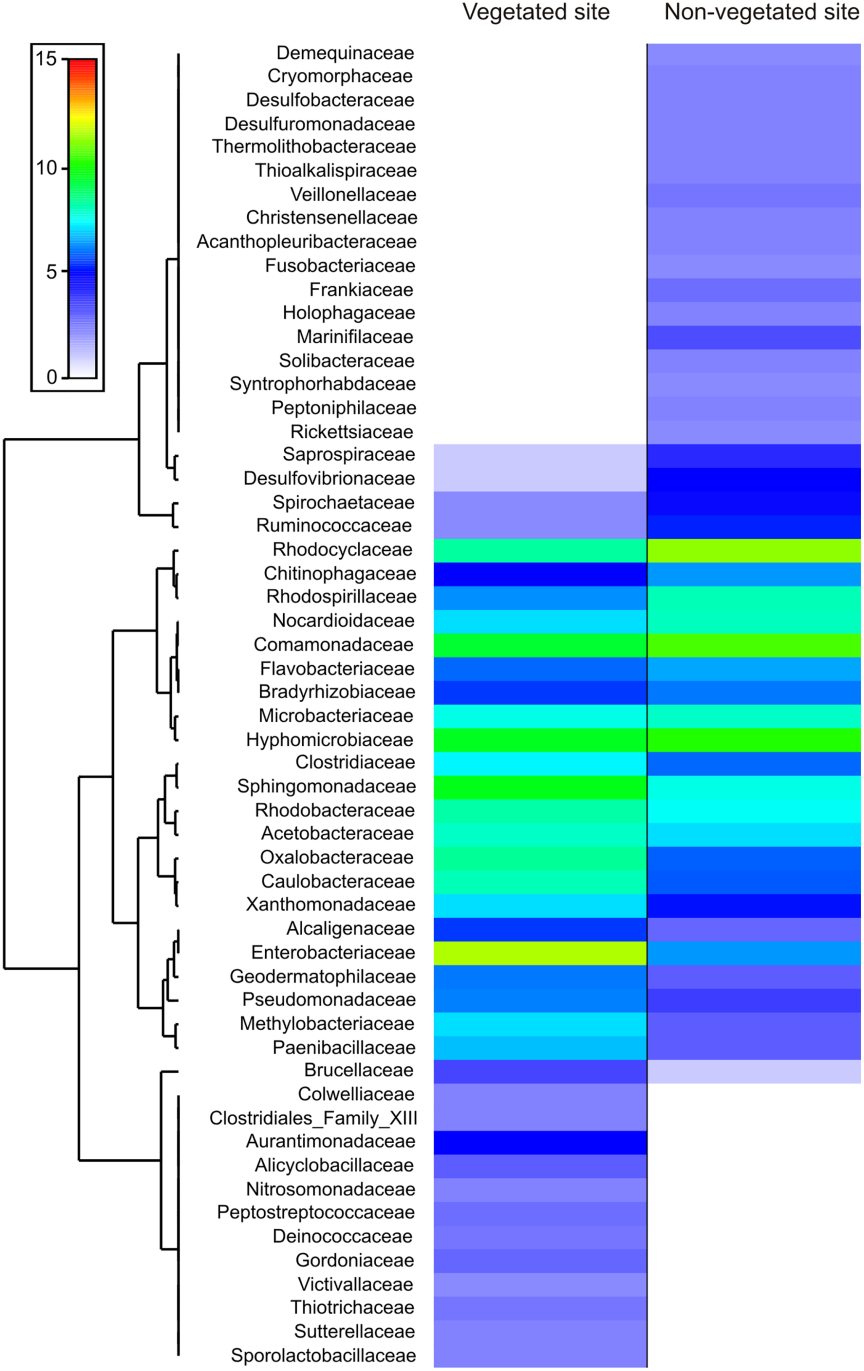
Families that contribute to bacterial dissimilarity. Families that contributed to bacterial dissimilarity and dominance between vegetated (V) and non-vegetated (NV) sites. First column: vegetated sites. Second column: non-vegetated sites.

The dominance analysis and SIMPER outputs showed that 56 families contributed mostly to bacterial dissimilarity and dominance between the vegetated (V) and non-vegetated (NV) sites (Fig. 4, Fig. S1, Table S2). Of these, 19 families contributed ~40% of the accumulated relative abundance (dominance) in both sites: Acetobacteraceae, Bradyrhizobiaceae, Caulobacteraceae, Chitinophagaceae, Clostridiaceae, Comamonadaceae, Enterobacteriaceae, Flavobacteriaceae, Hyphomicrobiaceae, Methylobacteriaceae, Microbacteriaceae, Nocardioidaceae Oxalobacteraceae, Rhodobacteraceae, Rhodocyclaceae, Rhodospirillaceae, Sphingomonadaceae, Veillonellaceae and Xanthomonadaceae (Fig. 4). Eight families were dominant, with similar relative abundance at both sites, and did not contribute significantly to the dissimilarity between sites (Bradyrhizobiaceae, Chitinophagaceae, Clostridiaceae, Comamonadaceae, Flavobacteriaceae, Rhodobacteraceae, Rhodospirillaceae, and Sphingomonadaceae). Another five families presented a different dominance and contribute to the dissimilarity between sites (Caulobacteraceae, Enterobacteriaceae, Methylobacteriaceae, Oxalobacteraceae, and Rhodocyclaceae, Fig. 4).

The families exclusive to each site presented low relative abundance values, but contributed to the dissimilarity between sites (Fig. 4). Twelve families were recorded exclusively in the vegetated site, (Alicyclobacillaceae, Aurantimonadaceae, Clostridiales_Family_XIII, Colwelliaceae, Deinococcaceae, Gordoniaceae, Nitrosomonadaceae, Peptostreptococcaceae, Sporolactobacillaceae, Sutterellaceae, Thiotrichaceae, and Victivallaceae) and 17 exclusively in the non-vegetated site (Acanthopleuribacteraceae, Christensenellaceae, Cryomorphaceae, Demequinaceae, Desulfobacteraceae, Desulfuromonadaceae, Frankiaceae, Fusobacteriaceae, Holophagaceae, Marinifilaceae, Peptoniphilaceae, Rickettsiaceae, Solibacteraceae, Syntrophoriolaceaceae, Theribacteraceae, Thioalkalispiraceae, and Veillonellaceae) (Fig. 4).

The putative bacterial functional diversity of the 218 families recorded was classified into 44 categories (Table S3). The predominant functions at the two analysis levels (shared, vegetated and non-vegetated) were aerobic chemoheterotrophy and fermentation. The main functions performed by the shared families were aerobic chemoheterotrophy (28%), fermentation (20%), ureolysis (7%), nitrate reduction (6%), and cellulolysis (6%). Another 39 functions were present but at lower percentages (Table S1). In *P. grandis*, there are minimal differences in the percentage of the main metabolic functions performed in vegetated and non-vegetated sites. In the vegetated site, aerobic chemoheterotrophy (27%), fermentation (20%), cellulolysis (7%), and ureolysis (6%) were the most important functions. Finally, the main metabolic functions in the non-vegetated site were aerobic chemoheterotrophy (27%), fermentation (23%), ureolysis (7%), and nitrate reduction (6%) (Table S1).

**Figure 5:**
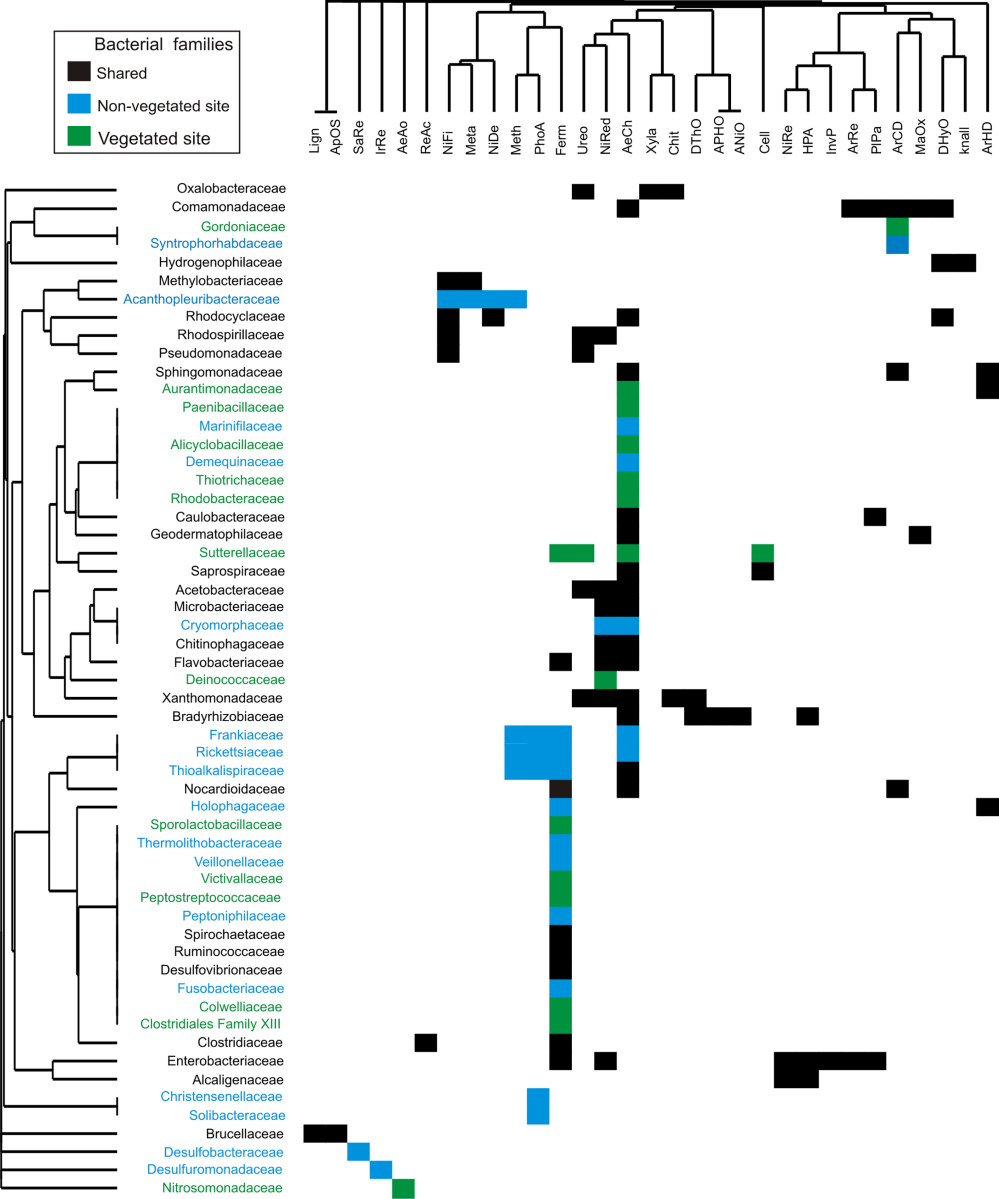
Bacterial families from vegetated and non-vegetated sites. The most important bacterial families that contributed to dominance and dissimilarity between sites and their putative metabolic functions. Black: present at both sites. Blue: present at non-vegetated sites only. Green: present at vegetated sites only.

The 56 bacterial families identified by dominance and SIMPER analysis perform 31 putative metabolic functions (Fig. 5). The dominant bacterial families with most functions were Enterobacteriaceae (7), Comamonadaceae (6), Bradyrhizobiaceae (5), and Xanthomonadaceae (5) (Fig. 5). The predominant functions in both sites were aerobic chemoheterotrophy (46%), and fermentation (38%), carried out by 26 and 21 families, respectively. Other functions, such as nitrate reduction (16 %), ureolysis (11%), aromatic compound degradation, and nitrogen fixation (9%), were performed by less than nine families. Aerobic ammonia oxidation was exclusively found in the vegetated site related to the family Nitrosomonadaceae. Four functions were present exclusively in the non-vegetated site: iron and sulfur respiration (1%) performed by Desulfuromonadaceae and Desulfobacteraceae respectively. Photoautotrophy (9%) and methylotrophy (7%) were performed by Frankiaceae, Rickettsiaceae and Thioalkalispiraceae, while Christensenellaceae only participated in photoautotrophy and Acanthopleuribacteraceae in methylotrophy (Fig. 5).

## Discussion

The results of this study indicate that the composition of bacterial families in the phytotelmata of *P. grandis* is similar between the vegetated and non-vegetated sites. Nevertheless, they present a different dominance pattern as a function of the richness of OTUs associated with these families. Bacterial richness in *P. grandis* is composed of 23 phyla and 218 families. This result contrasts with *Aechmea bromeliifolia* and *A. nudicaulis*, each of which contain 51 phyla (Rodríguez-Nuñez, Rullan-Cardec & Rios-Velazquez, 2018), and with the 30 phyla reported in *Aechmea gamosepala, Vriesea friburgensis*, and *V. platynema* (Simão et al., 2020). However, at the family level, we found a greater richness in *P. grandis* compared to *Aechmea nudicaulis* (81 families Louca et al., 2016), *A. nudicaulis* and *Neoregelia cruenta* (56 families Louca et al., 2017), and *A. gamosepala* and *V. platynema* (103 families, Giongo et al., 2019). We considered that the families with a low richness of OTUs that contribute a low percentage (<1%) to the diversity of *P. grandis* could possibly be considered as a rare biosphere (Pedrós-Alió, 2012; Jousset et al., 2017). Although the record of these families could be a product of chance rather than ecological forces, the triplicate sequencing decreases such probability. In this study, another factor contributing to the detection of these OTUs was the use of seven hypervariable regions of the 16S. Some studies demonstrate that these regions vary in sensitivity and level of informativeness for different approaches (Yang, Wang & Qian, 2016; Fiannaca et al., 2018; Huttenhower et al., 2012; Soergel et al., 2012; D’Amore et al., 2016; Zheng et al., 2015; Chakravorty et al., 2007).

The dominant metabolic function within the bacterial community in tank bromeliad is the decomposition of complex organic compounds accumulated as vegetal detritus (Louca et al., 2016). Members of the Phylum Actinobacteria are saprophytes that decompose a wide spectrum of plant and animal remains (Zhang et al., 2017). They also occur in polluted environments of both terrestrial and aquatic ecosystems (Rosenberg, Delong & Thompson, 2014). Proteobacteria are the dominant group in soil microbial communities (Zhang et al., 2017), as well as in bromeliad phytotelmata (Louca et al., 2016; Louca et al., 2017). Many Firmicutes can also decompose organic debris, resist high temperatures, and remain in dehydrated environments by inactivity (Parkes & Sass, 2009). Their presence in the *P. grandis* tanks suggests the occurrence of a nutrient recycling process, which provides resources for both the associated biota and the plant itself.

Despite some differences in bacterial taxonomic diversity in *P. grandis* between vegetated and non-vegetated sites, the dominant bacteria share most of the metabolic functions. The six main functions, aerobic chemoheterotrophy, fermentation, ureolysis, nitrate reduction, aromatic compound degradation, and nitrogen fixation, are prominent, since these are carried out by the families with a greater amount of OTUs in both sites. The first three functions mentioned above occur in equal percentages when the 212 families were included. *Aechmea nudicaulis* (L.) Griseb. (Bromeliaceae) and *Sarracenia purpurea* L. (Sarraceniaceae) present different bacterial composition in their phytotelmata, but with similar functions (Louca et al., 2016; Grothjan & Young, 2019). However, when the geochemical conditions of the tanks of *A. nudicaulis* and *Neoregelia cruenta* (Graham) L.B. Sm. (Bromeliaceae) are compared, functional community structure is strongly correlated with the different ecological conditions provided by the vegetal cover and access to freshwater (Louca et al., 2017). In *P. grandis* when the total family richness is considered a slight decrease in cellulolysis (7% *vs*. 5%) and a slight increase in fermentation (21% *vs*. 23%) were detected in vegetated compared to non-vegetated sites. Moreover, some putative functions were recorded only in one site and related to families with the lowest richness of OTUs. These differences could be related to environmental factors that were not considered in this study. More studies are required to gather conclusive evidence in this regard.

The bacterial composition of *P. grandis* shows differences between sites in terms of the exclusive families, relative abundance of OTUs, and percentages of putative metabolic functions performed. Although the two sites share 68.3% of their composition, the unshared families suggest variations in the physiochemical conditions of the phytotelmata at each site. The bacterial community of vegetated site presents families which require an acidic pH and high levels of organic carbon and nitrogen compounds. For example, Alicyclobacillaceae grows in acid environments produced by carbohydrates (Stackebrandt, 2014). Deinococcaeae can live with high radiation levels (Murray, 1992), and Nitrosomonadaceae play significant roles in control of the nitrogen cycle in freshwater environments as ammonia oxidizers (Prosser, Head & Stein, 2014) (Fig. 4). In contrast, the sample from the non-vegetated site contained families with metabolic functions that are associated with autotrophic organisms, and others adapted to carbon and oxygen scarcity that utilize inorganic nitrogen and sulfur compounds deposited by rock sediments in their life cycle. Some of the families are Fusobacteriaceae that ferment carbohydrates and can live in anaerobic sediments (Olsen, 2014). Desulfobacteraceae are strictly anaerobic sulfate-reducing bacteria that grow best at moderate temperatures (Kuever, 2014). Desulfuromonadaceae are found in anoxic environments and are associated with methanogens and phototrophic green sulfur bacteria (Greene, 2014) (Figs. 4, 5). The families Frankiaceae, Rickettsiaceae, and Thioalkalispiraceae also perform methylotrophy (*i.e*. they can obtain energy from single-carbon compounds). The largest number of families belongs to the orders Actinomycetales and Rhizobiales, taxa that degrade plant debris and comprise genera (such as *Streptomyces* and *Rhizobium*) that present symbiotic relationships with plants. Their function in *P. grandis* is as degraders and symbionts, promoting plant growth and maintaining the ecosystem formed inside the bromeliad. The differences in the orders and families of bacteria unique to each site indicate that, when the phytotelma is exposed, the biota will mostly be autotrophic and will utilize the rock sediments from the slope (chemoautotrophs) and sunlight (phototrophs) for their metabolic functions.

The bacterial diversity found in the tank suggests that the organisms that inhabit these small aquatic microhabitats take advantage of water availability to develop. After the dry season, endospores in the tank, or from the environment around the tank (*e.g*., in the air, in the debris) proliferate quickly during the short rainy season and are specialized in the decomposition of complex organic compounds. Rare biosphere bacteria (OTUs or species with frequencies less than or equal to 1% (Pedrós-Alió, 2012)) play important ecological roles as drivers of ecosystem key functions. They are also considered genetic reservoirs, the abundance of which depends on external abiotic and biotic factors. Their interactions could favor micro-ecosystem resilience and resistance (Coveley, Elshahed & Youssef, 2015; Jousset et al., 2017). We found that a few families also present in low frequencies have putative metabolic functions recorded for one site only. They include Alcanivoracaceae, which are involved in aliphatic non-methane hydrocarbon degradation and oil bioremediation. However, the presence of these families should be treated with some caution. Future studies on tank bromeliads should address the relationship between the rare families and the maintenance of the micro-ecosystem.

## Conclusions

We hypothesized that bacterial diversity in the phytotelmata from an arid zone would differ in sites with and without surrounding vegetation. Slight differences were found for *Pseudalcantarea grandis* in taxonomic richness, number of OTUs for the dominant and exclusive families, and the putative metabolic functions performed in each site. The non-vegetated site was richer in families and exclusive OTUs than the vegetated site. In the latter, families such as Deinococcaeae and Nitrosomonodaceae prefer an acidic pH and high levels of nutrients. The phytotelma of the non-vegetated site contain families such as Fusobacteriaceae and Desulfobacteraceae that thrive under carbon and oxygen shortage and can metabolize inorganic and sulfur compounds. The organisms that inhabit the small ephemeral aquatic microhabitats are well adapted to prolonged dry periods and development quickly in water presence. Their taxonomic variation could fulfill specialized functions in the degradation of organic matter, photo- or chemoautotrophy depending on the exposure to different conditions. Our study is the first to characterize the *P. grandis* microbiome and the information generated will be of utility to new studies in tank bromeliads and related groups.

## Supplemental Information

10.7717/peerj.12706/supp-1 Supplemental Information 1 Bacterial diversity for vegetated and unvegetated sites. Bacterial diversity by taxonomic order for the vegetated and unvegetated sites. Composed by 23 Phyla, 52 Classes, 98 Orders, and 218 Families. The number of families of each order is shown for each site.

10.7717/peerj.12706/supp-2 Supplemental Information 2 Similarity percentage analysis (SIMPER) results of the bacterial families between vegetated (V) and non-vegetated (NV) sites considering a cumulative contribution of ~40% and number of OTUS by family. Results of the bacterial families between vegetated (V) and non-vegetated (NV) sites considering a cumulative contribution of ~40% and number of OTUS by family

10.7717/peerj.12706/supp-3 Supplemental Information 3 List of putative bacterial functions in the *P. grandis* tank. Putative bacterial functional diversity identified in *Pseudalcantarea grandis* (Bromeliaceae)

10.7717/peerj.12706/supp-4 Supplemental Information 4 Rank/abundance curves of bacterial families in vegetated (V) and non-vegetated (NV) sites. The graphs show bacterial family dominance in vegetated (V) and non-vegetated (NV) sites

## Additional Information and Declarations

### Field Study Permissions

The following information was supplied relating to field study approvals (*i.e*., approving body and any reference numbers): Field sampling was approved by the Facultad de Ciencias Naturales bioethics committee (39FCN2019).

### Data Availability

The following information was supplied regarding data availability: The sequences of the seven hypervariable regions of the 16S are available at NCBI PRJNA685432.

## References

[ref-1] Arévalo V (1991). Excavación de galerías de inyección, drenaje e inspección del proyecto 3 hidroeléctrico zimapán. Boletín de la Sociedad Geológica Mexicana.

[ref-2] Benzing DH (2000). Bromeliaceae: profile of an adaptive radiation.

[ref-3] Bergey DH, Holt JG (2005). Bergey’s manual of determinative bacteriology.

[ref-4] Brandt FB, Martinson GO, Conrad R (2016). Bromeliad tanks are unique habitats for microbial communities involved in methane turnover. Plant and Soil.

[ref-5] Brozio S, Manson C, Gourevitch E, Burns TJ, Greener MS, Downie JR, Hoskisson PA (2017). Development and application of an eDNA method to detect the critically endangered Trinidad golden tree frog (*Phytotriades auratus*) in bromeliad phytotelmata. PLOS ONE.

[ref-6] Cabral BCA, Hoffmann L, Budowle B, Ürményi TP, Moura-Neto RS, Azevedo SMFO, Silva R (2018). Planktonic microbial profiling in water samples from a Brazilian Amazonian reservoir. MicrobiologyOpen.

[ref-7] Calhoun AJK, Mushet DM, Bell KP, Boix D, Fitzsimons JA, Isselin-Nondedeu F (2017). Temporary wetlands: challenges and solutions to conserving a disappearing’ ecosystem. Biological Conservation.

[ref-8] Carrillo M (1981). Contribución al estudio geológico del macizo calcáreo El Doctor, Querétaro. Revista Mexicana de Ciencias Geológicas.

[ref-9] Carrillo M, Sutter M (1981). Tectónica de los alrededores de Zimapán, Hidalgo y Querétaro. Guía de la excursión geológica a la Región Zimapán y áreas circundantes.

[ref-47] Chakravorty S, Helb D, Burday M, Connell N, Alland DA (2007). A detailed analysis of 16S ribosomal RNA gene segments for the diagnosis of pathogenic bacteria. Journal of Microbiological Methods.

[ref-10] Clarke KR, Gorley RN (2015). Primer v7: user manual/tutorial.

[ref-11] Coveley S, Elshahed MS, Youssef NH (2015). Response of the rare biosphere to environmental stressors in a highly diverse ecosystem (Zodletone spring. OK, USA). PeerJ.

[ref-45] D’Amore R, Ijaz UZ, Schirmer M, Kenny JG, Gregory R, Darby AC, Shakia M, Podar M, Quince C, Hall N (2016). A comprehensive benchmarking study of protocols and sequencing platforms for 16S rRNA community profiling. BMC Genomics.

[ref-42] Fiannaca A, La Paglia L, La Rosa M, Lo Bosco G, Renda G, Rizzo R, Gaglio S, Urso A (2018). Deep learning models for bacteria taxonomic classification of metagenomic data. BMC Bioinformatics.

[ref-12] Giongo A, Medina-Silva R, Astarita LV, Borges LG, Oliveira RR, Simão TLL, Eizirik E (2019). Seasonal physiological parameters and phytotelmata bacterial diversity of two bromeliad species (*Aechmea gamosepala* and *Vriesea platynema*) from the Atlantic Forest of Southern Brazil. Diversity.

[ref-13] Goffredi SK, Jang GE, Haroon MF (2015). Transcriptomics in the tropics: total RNA-based profiling of Costa Rican bromeliad-associated communities. Computational and Structural Biotechnology Journal.

[ref-14] Goffredi SK, Kantor AH, Woodside WT (2011). Aquatic microbial habitats within a neotropical rainforest: Bromeliads and pH-associated trends in bacterial diversity and composition. Microbial Ecology.

[ref-15] Greene AC, Rosenberg E, DeLong EF, Lory S, Stackebrandt E, Thompson F (2014). The family desulfuromonadaceae. The Prokaryotes.

[ref-16] Grothjan JJ, Young EB (2019). Diverse microbial communities hosted by the model carnivorous pitcher plant *Sarracenia purpurea*: analysis of both bacterial and eukaryotic composition across distinct host plant populations. PeerJ.

[ref-17] Hernández HM, Gómez-Hinostrosa C, Cartron JL, Ceballos G, Felger R (2005). Cactus diversity and endemism in the Chihuahuan Desert Region. Biodiversity, Ecosystems, and Conservation in Northern Mexico.

[ref-43] Huttenhower C, Gevers D, Knight R, Abubucker S, Badger JH, Chinwalla AT, The Human Microbiome Project Consortium (2012). Structure, function and diversity of the healthy human microbiome. Nature.

[ref-18] Jousset A, Bienhold C, Chatzinotas A, Gallien L, Gobet A, Kurm V (2017). Where less may be more: how the rare biosphere pulls ecosystems strings. The ISME Journal.

[ref-19] Kitching RL (2001). Food webs in phytotelmata: “Bottom-Up” and “Top-Down” explanations for community structure. Annual Review of Entomology.

[ref-20] Kuever J, Rosenberg E, DeLong EF, Lory S, Stackebrandt E, Thompson F (2014). The family desulfobacteraceae. The Prokaryotes.

[ref-21] Louca S, Jacques SMS, Pires APF, Leal JS, González AL, Doebeli M, Farjalla VF (2017). Functional structure of the bromeliad tank microbiome is strongly shaped by local geochemical conditions. Environmental Microbiology.

[ref-22] Louca S, Jacques SMS, Pires APF, Leal JS, Srivastava DS, Parfrey LW, Doebeli M (2016). High taxonomic variability despite stable functional structure across microbial communities. Nature Ecology and Evolution.

[ref-23] Louca S, Parfrey LW, Doebeli M (2016). Decoupling function and taxonomy in the global ocean microbiome. Science.

[ref-24] Males J (2016). Think tank: water relations of Bromeliaceae in their evolutionary context. Botanical Journal of the Linnean Society.

[ref-25] Medellín-Leal F, Bender GL (1982). The chihuahuan desert. Reference Handbook on the Deserts of North America.

[ref-26] Mogi M (2004). Phytotelmata: hidden freshwater habitats supporting unique faunas. Freshwater Invertebrates of the Malaysian Region.

[ref-27] Murray RGE, Balows A, Trüper HG, Dworkin M, Harder W, Schleifer KH (1992). The family deinococcaceae. The Prokaryotes.

[ref-28] Olsen I, Rosenberg E, DeLong EF, Lory S, Stackebrandt E, Thompson F (2014). The family fusobacteriaceae. The Prokaryotes.

[ref-29] Parkes RJ, Sass H, Schnaechter M (2009). Deep sub surface. Encyclopedia of Microbiology.

[ref-30] Pedrós-Alió C (2012). The rare bacterial biosphere. Annual Review of Marine Sciences.

[ref-31] Prosser JI, Head IM, Stein LY, Rosenberg E, DeLong EF, Lory S, Stackebrandt E, Thompson F (2014). The family nitrosomonadaceae. The Prokaryotes.

[ref-32] R Core Team (2019). R: a language and environment for statistical computing. R foundation for statistical computing, Vienna, Austria. https://www.R-project.org/.

[ref-33] Rodríguez-Nuñez KM, Rullan-Cardec JM, Rios-Velazquez C (2018). The metagenome of bromeliads phytotelma in Puerto Rico. Data in Brief.

[ref-34] Rosenberg E, Delong EF, Thompson F, Fourth E, Rosenberg EF, Delong EF, Thompson F (2014). The phylum Actinobacteria. The Prokaryotes: Firmicutes and Tenericutes.

[ref-35] Rzedowski J (2006). Vegetación de México.

[ref-36] Segestrom K (1961). Geología del sureste del estado de Hidalgo y del noreste del estado de México. Boletín de la Asociación Mexicana de Geólogos Petroleros.

[ref-37] Simão TLL, Borges AG, Gano KA, Davis-Richardson AG, Brown CT, Fagen JR, Triplett EW, Dias R, Mondin CA, da Silva RM, Eizirik E, Utz LRP (2017). Characterization of ciliate diversity in bromeliad tank waters from the Brazilian Atlantic Forest. European Journal of Protistology.

[ref-38] Simão TLL, Utz LRP, Dias R, Giongo A, Triplett EW, Eizirik E (2020). Remarkably complex microbial community composition in bromeliad tank waters revealed by eDNA metabarcoding. Journal of Eucaryotic Microbiology.

[ref-44] Soergel DA, Dey N, Knight R, Brenner SE (2012). Selection of primers for optimal taxonomic classification of environmental 16S rRNA gene sequences. ISME Journal.

[ref-39] Stackebrandt E, Rosenberg E, DeLong EF, Lory S, Stackebrandt E, Thompson F (2014). The family alicyclobacillaceae. The Prokaryotes.

[ref-41] Yang B, Wang Y, Qian PY (2016). Sensitivity and correlation of hypervariable regions in 16S rRNA genes in phylogenetic analysis. BMC Bioinformatics.

[ref-40] Zhang Z, Qu Y, Li S, Feng K, Wang S, Cai W, Liang Y, Li H, Xu M, Yin H, Deng Y (2017). Soil bacterial quantification approaches coupling with relative abundances reflecting the changes of taxa. Scientific Reports.

[ref-46] Zheng W, Tsompana M, Ruscitto A, Sharma A, Genco R, Sun Y, Buck JM (2015). An accurate and efficient experimental approach for characterization of the complex oral microbiota. Microbiome.

