# Supplementary material for: Metabarcoding of the phytotelmata of Pseudalcantarea grandis (Bromeliaceae) from an arid zone
Source: PeerJ. 2022 Jan 27;10:e12706. doi: 10.7717/peerj.12706 (PMC8801176; doi:10.7717/peerj.12706)
Supplement: Supplemental Information 1 — Bacterial diversity by taxonomic order for the vegetated and unvegetated sites. Composed by 23 Phyla, 52 Classes, 98 Orders, and 218 Families. The number of families of each order is shown for each site. [file peerj-10-12706-s001.docx]

Table S1. Bacterial diversity by taxonomic order for the vegetated and unvegetated sites. Composed by 23 Phyla, 52 Classes, 98 Orders, and 218 Families. The number of families of each order is shown for each site.

|  |  |  | Families | |
| --- | --- | --- | --- | --- |
| Phylum | Class | Order | Vegetated | Unvegetated |
| Acidobacteria | Acidobacteriia | Acidobacteriales | 1 | 2 |
| Acidobacteria | Holophagae | Acanthopleuribacterales | - | 1 |
| Acidobacteria | Holophagae | Holophagales | - | 1 |
| Acidobacteria | Solibacteres | Solibacterales | - | 1 |
| Actinobacteria | Acidimicrobiia | Acidimicrobiales | 3 | 3 |
| Actinobacteria | Actinobacteria | Actinomycetales | 23 | 28 |
| Actinobacteria | Actinobacteria | Bifidobacteriales | - | 1 |
| Actinobacteria | Actinobacteria | Euzebyales | 1 | 1 |
| Actinobacteria | Actinobacteria | Gaiellales | 1 | 1 |
| Actinobacteria | Actinobacteria | Nitriliruptorales | 1 | 1 |
| Actinobacteria | Actinobacteria | Rubrobacterales | 1 | 1 |
| Actinobacteria | Actinobacteria | Solirubrobacterales | 3 | 3 |
| Actinobacteria | Coriobacteriia | Coriobacteriales | 1 | 1 |
| Actinobacteria | Thermoleophilia | Thermoleophilales | 1 | 1 |
| Aquificae | Aquificae | Desulfurobacteriales | 1 | - |
| Armatimonadetes | Armatimonadia | Armatimonadales | - | 1 |
| Armatimonadetes | Chthonomonadetes | Chthonomonadales | - | 1 |
| Bacteroidetes | [Saprospirae] | [Saprospirales] | - | 1 |
| Bacteroidetes | Bacteroidia | Bacteroidales | 4 | 6 |
| Bacteroidetes | Cytophagia | Cytophagales | 2 | 2 |
| Bacteroidetes | Flavobacteriia | Flavobacteriales | 1 | 2 |
| Bacteroidetes | Sphingobacteriia | Sphingobacteriales | 3 | 3 |
| Chlamydiae | Chlamydiia | Chlamydiales | 5 | 4 |
| Chlorobi | Chlorobia | Chlorobiales | - | 1 |
| Chloroflexi | Anaerolineae | Anaerolineales | 1 | 1 |
| Chloroflexi | Anaerolineae | Ardenscatenales | 1 | 1 |
| Chloroflexi | Ardenticatenia | Ardenticatenales | 1 | - |
| Chloroflexi | Caldilineae | Caldilineales | 1 | 1 |
| Chloroflexi | Chloroflexia | Chloroflexales | 1 | - |
| Chloroflexi | Dehalococcoidia | Dehalococcoidales | 1 | 1 |
| Chloroflexi | Ktedonobacteria | Ktedonobacterales | 2 | 1 |
| Chloroflexi | Thermomicrobia | Sphaerobacterales | 1 | 1 |
| Cyanobacteria | Gloeobacterophycideae | Gloeobacterales | 1 | 1 |
| Cyanobacteria | Nostocophycideae | Nostocales | 1 | 1 |
| Cyanobacteria | Oscillatoriophycideae | Chroococcales | 1 | 1 |
| Cyanobacteria | Oscillatoriophycideae | Oscillatoriales | 1 | 1 |
| Deinococcus-Thermus | Deinococci | Deinococcales | 1 | - |
| Deinococcus-Thermus | Deinococci | Thermales | 1 | - |
| Firmicutes | Bacilli | Bacillales | 6 | 5 |
| Firmicutes | Bacilli | Lactobacillales | 5 | 5 |
| Firmicutes | Clostridia | Clostridiales | 11 | 12 |
| Firmicutes | Clostridia | Thermoanaerobacterales | 1 | 1 |
| Firmicutes | Erysipelotrichi | Erysipelotrichales | - | 1 |
| Firmicutes | Negativicutes | Selenomonadales | 1 | 1 |
| Firmicutes | Thermolithobacteria | Thermolithobacterales | - | 1 |
| Fusobacteria | Fusobacteriia | Fusobacteriales | - | 1 |
| Gemmatimonadetes | Gemmatimonadetes | Gemmatimonadales | 1 | 1 |
| Ignavibacteriae | Ignavibacteria | Ignavibacteriales | 1 | 2 |
| Lentisphaerae | Lentisphaeria | Victivallales | 1 | - |
| Nitrospinae | Nitrospinia | Nitrospinales | 1 | 1 |
| Nitrospirae | Nitrospira | Nitrospirales | 2 | 2 |
| Planctomycetes | Phycisphaerae | Phycisphaerales | 1 | 1 |
| Planctomycetes | Planctomycetia | Candidatus Brocadiales | - | 1 |
| Planctomycetes | Planctomycetia | Planctomycetales | 1 | 1 |
| Proteobacteria | Alphaproteobacteria | Caulobacterales | 1 | 1 |
| Proteobacteria | Alphaproteobacteria | Rhizobiales | 12 | 10 |
| Proteobacteria | Alphaproteobacteria | Rhodobacterales | 2 | 2 |
| Proteobacteria | Alphaproteobacteria | Rhodospirillales | 2 | 2 |
| Proteobacteria | Alphaproteobacteria | Rickettsiales | 1 | 1 |
| Proteobacteria | Alphaproteobacteria | Sneathiellales | 1 | - |
| Proteobacteria | Alphaproteobacteria | Sphingomonadales | 2 | 2 |
| Proteobacteria | Betaproteobacteria | Burkholderiales | 5 | 4 |
| Proteobacteria | Betaproteobacteria | Ferritrophicales | 1 | 1 |
| Proteobacteria | Betaproteobacteria | Gallionellales | 1 | 1 |
| Proteobacteria | Betaproteobacteria | Hydrogenophilales | 1 | 1 |
| Proteobacteria | Betaproteobacteria | Methylophilales | - | 1 |
| Proteobacteria | Betaproteobacteria | Neisseriales | 2 | 2 |
| Proteobacteria | Betaproteobacteria | Nitrosomonadales | 1 | - |
| Proteobacteria | Betaproteobacteria | Rhodocyclales | 1 | 1 |
| Proteobacteria | Betaproteobacteria | Sulfuricellales | 1 | 1 |
| Proteobacteria | Deltaproteobacteria | Bdellovibrionales | 2 | 1 |
| Proteobacteria | Deltaproteobacteria | Desulfobacterales | 1 | 2 |
| Proteobacteria | Deltaproteobacteria | Desulfovibrionales | 2 | 2 |
| Proteobacteria | Deltaproteobacteria | Desulfurellales | 1 | 1 |
| Proteobacteria | Deltaproteobacteria | Desulfuromonadales | 2 | 3 |
| Proteobacteria | Deltaproteobacteria | Myxococcales | 6 | 5 |
| Proteobacteria | Deltaproteobacteria | Syntrophobacterales | 2 | 3 |
| Proteobacteria | Epsilonproteobacteria | Campylobacterales | 2 | 2 |
| Proteobacteria | Epsilonproteobacteria | Nautiliales | - | 1 |
| Proteobacteria | Gammaproteobacteria | Acidithiobacillales | 1 | 1 |
| Proteobacteria | Gammaproteobacteria | Aeromonadales | 1 | 1 |
| Proteobacteria | Gammaproteobacteria | Alteromonadales | 2 | 2 |
| Proteobacteria | Gammaproteobacteria | Arenicellales | 1 | - |
| Proteobacteria | Gammaproteobacteria | Chromatiales | 2 | 3 |
| Proteobacteria | Gammaproteobacteria | Enterobacteriales | 1 | 1 |
| Proteobacteria | Gammaproteobacteria | Legionellales | 2 | 2 |
| Proteobacteria | Gammaproteobacteria | Methylococcales | 1 | 1 |
| Proteobacteria | Gammaproteobacteria | Oceanospirillales | 3 | 2 |
| Proteobacteria | Gammaproteobacteria | Pasteurellales | 1 | - |
| Proteobacteria | Gammaproteobacteria | Pseudomonadales | 2 | 2 |
| Proteobacteria | Gammaproteobacteria | Thiotrichales | 2 | 1 |
| Proteobacteria | Gammaproteobacteria | Xanthomonadales | 2 | 2 |
| Spirochaetes | Spirochaetia | Spirochaetales | 3 | 3 |
| Synergistetes | Synergistia | Synergistales | - | 1 |
| Tenericutes | Mollicutes | Acholeplasmatales | 1 | 1 |
| Tenericutes | Mollicutes | Mycoplasmatales | 1 | - |
| Verrucomicrobia | Opitutae | Opitutales | 1 | 1 |
| Verrucomicrobia | Verrucomicrobiae | Verrucomicrobiales | 1 | 1 |
