# Supplementary material for: Metabarcoding of the phytotelmata of Pseudalcantarea grandis (Bromeliaceae) from an arid zone
Source: PeerJ. 2022 Jan 27;10:e12706. doi: 10.7717/peerj.12706 (PMC8801176; doi:10.7717/peerj.12706)
Supplement: Supplemental Information 2 — Results of the bacterial families between vegetated (V) and non-vegetated (NV) sites considering a cumulative contribution of ~40% and number of OTUS by family [file peerj-10-12706-s002.docx]

Metabarcoding of the phytotelmata of *Pseudalcantarea grandis* (Bromeliaceae) from an arid zone

José Alan Herrera-García^1^, Mahinda Martínez^1,3^, Pilar Zamora-Tavares^2,3^, Ofelia Vargas-Ponce^2,3^, Luis Hernández-Sandoval^1,3^, Fabián Alejandro Rodríguez-Zaragoza ^4^

Table Supplementary 2. Similarity percentage analysis (SIMPER) results of the bacterial families between vegetated (V) and non-vegetated (NV) sites considering a cumulative contribution of ~40% and number of OTUS by family

| Average dissimilarity = 25.26 | Group V | | Group NV | | Contrib% | Cum |
| --- | --- | --- | --- | --- | --- | --- |
| Family | Number of OTUS | | Number of OTUS | |  |  |
| Enterobacteriaceae | 136 | 41 | | 1.88 | | 1.88 |
| Aurantimonadaceae | 24 | 0 | | 1.76 | | 3.64 |
| Methylobacteriaceae | 50 | 10 | | 1.4 | | 5.04 |
| Desulfovibrionaceae | 1 | 24 | | 1.4 | | 6.44 |
| Paenibacillaceae | 46 | 10 | | 1.3 | | 7.73 |
| Marinifilaceae | 0 | 12 | | 1.24 | | 8.98 |
| Alicyclobacillaceae | 10 | 0 | | 1.13 | | 10.11 |
| Saprospiraceae | 1 | 17 | | 1.12 | | 11.23 |
| Gordoniaceae | 9 | 0 | | 1.07 | | 12.3 |
| Ruminococcaceae | 5 | 27 | | 1.06 | | 13.36 |
| Rhodocyclaceae | 70 | 128 | | 1.06 | | 14.42 |
| Geodermatophilaceae | 37 | 10 | | 1.05 | | 15.47 |
| Frankiaceae | 0 | 8 | | 1.01 | | 16.48 |
| Peptostreptococcaceae | 8 | 0 | | 1.01 | | 17.49 |
| Deinococcaceae | 7 | 0 | | 0.95 | | 18.44 |
| Veillonellaceae | 0 | 7 | | 0.95 | | 19.39 |
| Thiotrichaceae | 7 | 0 | | 0.95 | | 20.34 |
| Brucellaceae | 13 | 1 | | 0.93 | | 21.27 |
| Oxalobacteraceae | 71 | 34 | | 0.93 | | 22.2 |
| Spirochaetaceae | 5 | 23 | | 0.92 | | 23.12 |
| Alcaligenaceae | 30 | 9 | | 0.89 | | 24 |
| Acanthopleuribacteraceae | 0 | 6 | | 0.88 | | 24.88 |
| Holophagaceae | 0 | 6 | | 0.88 | | 25.76 |
| Solibacteraceae | 0 | 6 | | 0.88 | | 26.64 |
| Cryomorphaceae | 0 | 6 | | 0.88 | | 27.52 |
| Sporolactobacillaceae | 6 | 0 | | 0.88 | | 28.39 |
| Christensenellaceae | 0 | 6 | | 0.88 | | 29.27 |
| Clostridiales_Family_XIII | 6 | 0 | | 0.88 | | 30.15 |
| Peptoniphilaceae | 0 | 6 | | 0.88 | | 31.03 |
| Thermolithobacteraceae | 0 | 6 | | 0.88 | | 31.9 |
| Sutterellaceae | 6 | 0 | | 0.88 | | 32.78 |
| Nitrosomonadaceae | 6 | 0 | | 0.88 | | 33.66 |
| Desulfobacteraceae | 0 | 6 | | 0.88 | | 34.54 |
| Desulfuromonadaceae | 0 | 6 | | 0.88 | | 35.41 |
| Colwelliaceae | 6 | 0 | | 0.88 | | 36.29 |
| Thioalkalispiraceae | 0 | 6 | | 0.88 | | 37.17 |
| Pseudomonadaceae | 38 | 14 | | 0.87 | | 38.04 |
| Caulobacteraceae | 66 | 33 | | 0.85 | | 38.89 |
| Demequinaceae | 0 | 5 | | 0.8 | | 39.69 |
| Fusobacteriaceae | 0 | 5 | | 0.8 | | 40.49 |
| Victivallaceae | 5 | 0 | | 0.8 | | 41.29 |
| Rickettsiaceae | 0 | 5 | | 0.8 | | 42.1 |
| Syntrophorhabdaceae | 0 | 5 | | 0.8 | | 42.9 |

Notes: Group V= vegetated site, Group NV= Non-vegetated site, Contrib% = percentage contribution to dissimilarity, Cum= accumulated contribution
