# Supplementary material for: Metabarcoding of the phytotelmata of Pseudalcantarea grandis (Bromeliaceae) from an arid zone
Source: PeerJ. 2022 Jan 27;10:e12706. doi: 10.7717/peerj.12706 (PMC8801176; doi:10.7717/peerj.12706)
Supplement: Supplemental Information 3 — Putative bacterial functional diversity identified in Pseudalcantarea grandis (Bromeliaceae) [file peerj-10-12706-s003.docx]

Metabarcoding of the phytotelmata of *Pseudalcantarea grandis* (Bromeliaceae) from an arid zone

José Alan Herrera-García^1^, Mahinda Martínez^1,3^, Pilar Zamora-Tavares^2,3^, Ofelia Vargas-Ponce^2,3^, Luis Hernández-Sandoval^1,3^, Fabián Alejandro Rodríguez-Zaragoza ^4^

Table supplementary 3. The putative bacterial functional diversity identified in *Pseudalcantarea grandis* (Bromeliaceae)

| **Putative metabolic function** | **Code** |
| --- | --- |
| Aerobic ammonia oxidation | AeAO |
| Aerobic chemoheterotrophy | AeCh |
| Aerobic nitrite oxidation | ANi0 |
| Aliphatic non methane hydrocarbon degradation | AMHD |
| Anammox | Anam |
| Animal parasites or symbionts | APoS |
| Anoxygenic photoautotrophy | AnPh |
| Anoxygenic photoautotrophy H1 oxidizing | APHO |
| Anoxygenic photoautotrophy S oxidizing | APSO |
| Aromatic compound degradation | ArCD |
| Aromatic hydrocarbon degradation | ArHD |
| Arsenate respiration | ArRe |
| Cellulolysis | Cell |
| Chitinolysis | Chit |
| Dark hydrogen oxidation | DHyO |
| Dark iron oxidation | DIrO |
| Dark oxidation of sulfur compounds | DOSC |
| Dark thiosulfate oxidation | DThO |
| Fermentation | Ferm |
| Fumarate respiration | FuRe |
| Hydrocarbon degradation | HyDe |
| Intracellular parasites | IncP |
| Invertebrate parasites | InvP |
| Iron respiration | IrRe |
| Knallgas bacteria | knall |
| Ligninolysis | Lign |
| Manganese oxidation | MaOx |
| Methanotrophy | Meta |
| Methylotrophy | Meth |
| Nitrate denitrification | NiDe |
| Nitrate reduction | NiRed |
| Nitrate respiration | NiRe |
| Nitrogen fixation | NiFi |
| Nitrogen respiration | NiRe |
| Oil bioremediation | OilB |
| Photoautotrophy | PhoA |
| Photoheterotrophy | Phot |
| Plant pathogen | PlPa |
| Predatory or exoparasitic | PorE |
| Reductive acetogenesis | ReAc |
| Sulfate respiration | SuRe |
| Sulfur respiration | SaRe |
| Ureolysis | Ureo |
| Xylanolysis | Xyla |
