# Supplementary material for: Metabarcoding of the phytotelmata of Pseudalcantarea grandis (Bromeliaceae) from an arid zone
Source: PeerJ. 2022 Jan 27;10:e12706. doi: 10.7717/peerj.12706 (PMC8801176; doi:10.7717/peerj.12706)
Supplement: Supplemental Information 4 — The graphs show bacterial family dominance in vegetated (V) and non-vegetated (NV) sites [file peerj-10-12706-s004.docx]

Metabarcoding of the phytotelmata of *Pseudalcantarea grandis* (Bromeliaceae) from an arid zone

José Alan Herrera-García^1^, Mahinda Martínez^1,3^, Pilar Zamora-Tavares^2,3^, Ofelia Vargas-Ponce^2,3^, Luis Hernández-Sandoval^1,3^, Fabián Alejandro Rodríguez-Zaragoza ^4^


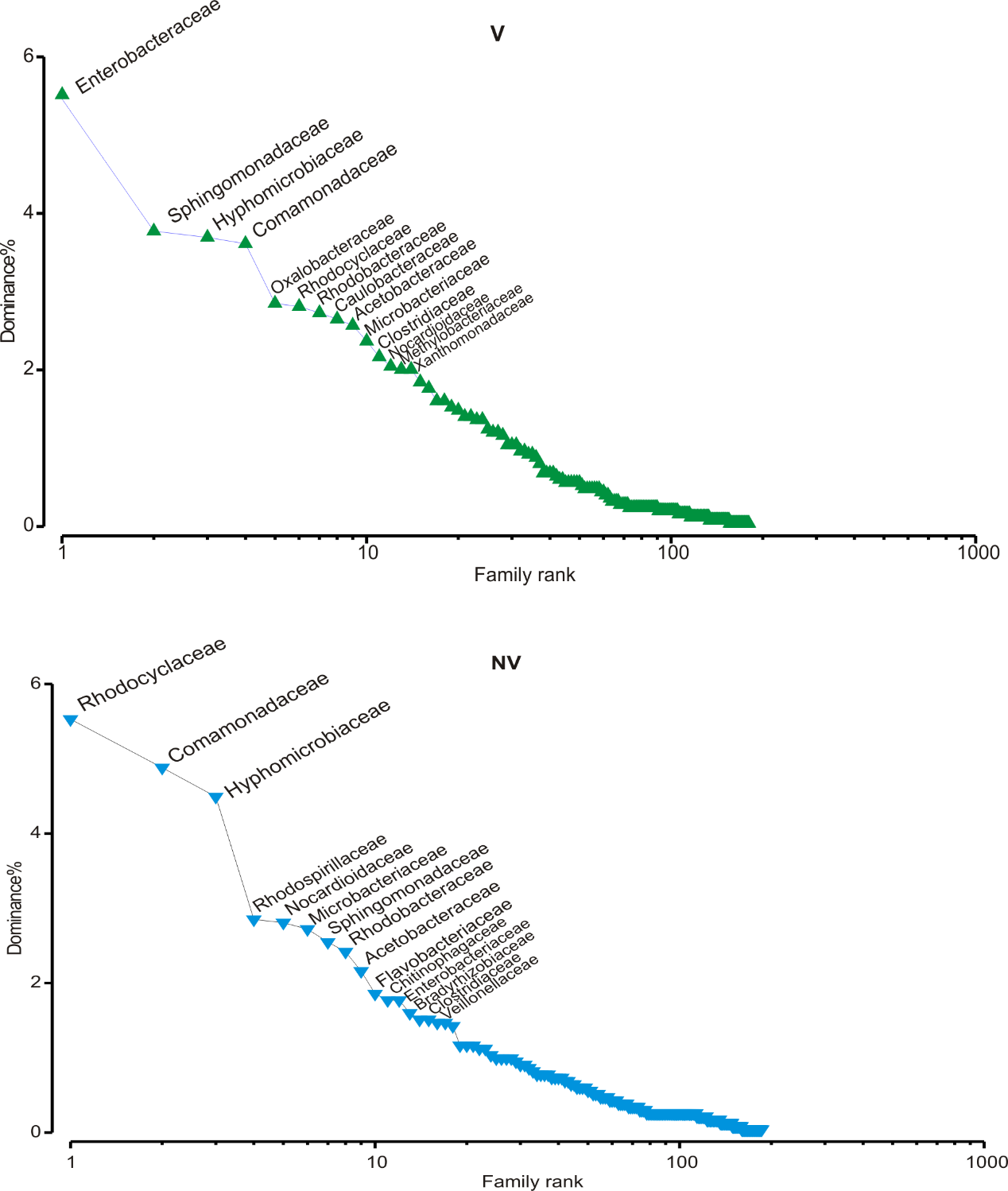


Figure supplementary 1. Rank/abundance curves of bacterial families in vegetated (V) and non-vegetated (NV) sites.
